# Supplementary material for: SARS-CoV-2 Specific Antibody Response and T Cell-Immunity in Immunocompromised Patients up to Six Months Post COVID: A Pilot Study
Source: J Clin Med. 2022 Jun 20;11(12):3535. doi: 10.3390/jcm11123535 (PMC9225567; doi:10.3390/jcm11123535)
Supplement: Supplementary file 1 [file jcm-11-03535-s001.zip › jcm-1689467-supplementary.pdf]

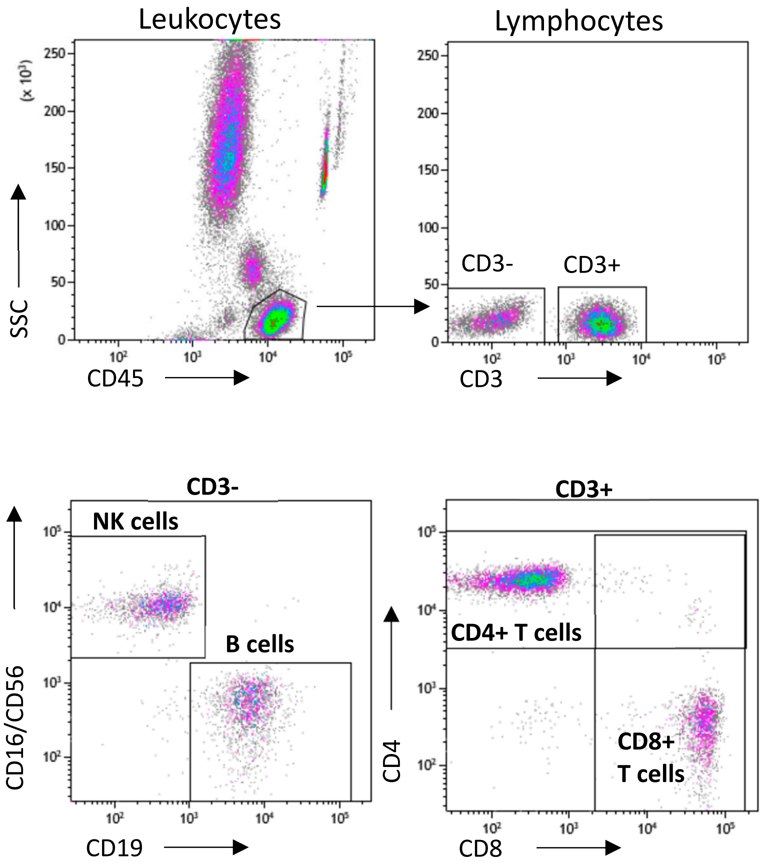

Figure S1. Gating strategy for CD4 T cells and CD8 T cells, B cells and NK cells.

a

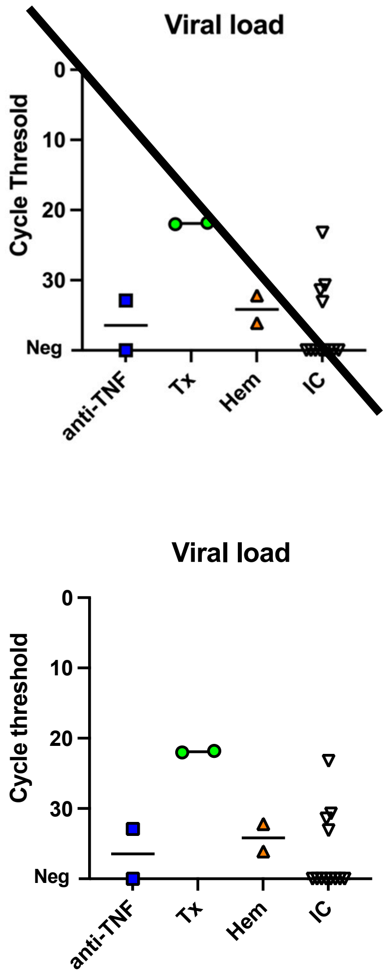
Viral load
Cycle Thresold
0
10
20
30
Neg
anti-TNF
Tx
Hem
IC
Viral load
Cycle threshold
0
10
20
30
Neg
anti-TNF
Tx
Hem
IC

b

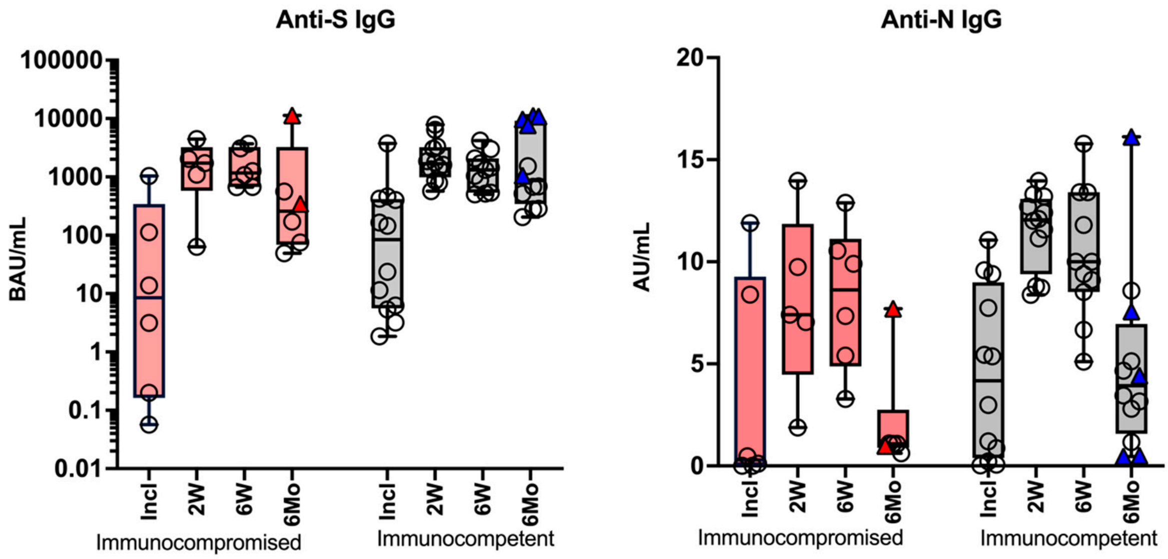
Anti-S IgG
BAU/mL
100000
10000
1000
100
10
1
0.1
0.01
Incl
2W
6W
6Mo
Incl
2W
6W
6Mo
Immunocompromised
Immunocompetent
Anti-N IgG
AU/mL
20
15
10
5
0
Incl
2W
6W
6Mo
Incl
2W
6W
6Mo
Immunocompromised
Immunocompetent

Figure S2. Detected SARS-CoV-2 RNA in nasopharyngeal samples in the study cohort at inclusion. The horizontal lines depict the median with negative samples given a cycle threshold value of 39 (half detection limit) (a). Anti-S (spike protein) IgG directed against the receptor binding domain and anti-N (nucleocapsid) IgG at inclusion (incl), two and six weeks, and six months after inclusion in immunocompromised and immunocompetent patients (b). Filled triangles depict patients that had received at least one dose of vaccine more than four weeks before sample collection. Cut-off for positive anti-S IgG was 14.2 BAU/mL and for anti-N IgG 1.4 AU/mL. Anti-TNF, patients on TNF inhibiting regimen (blue square); Tx, kidney transplant recipient (green circle); Hem, patients with myeloma or chronic lymphatic leukemia (orange triangle); BAU, binding antibody units; w, weeks; mo, months.

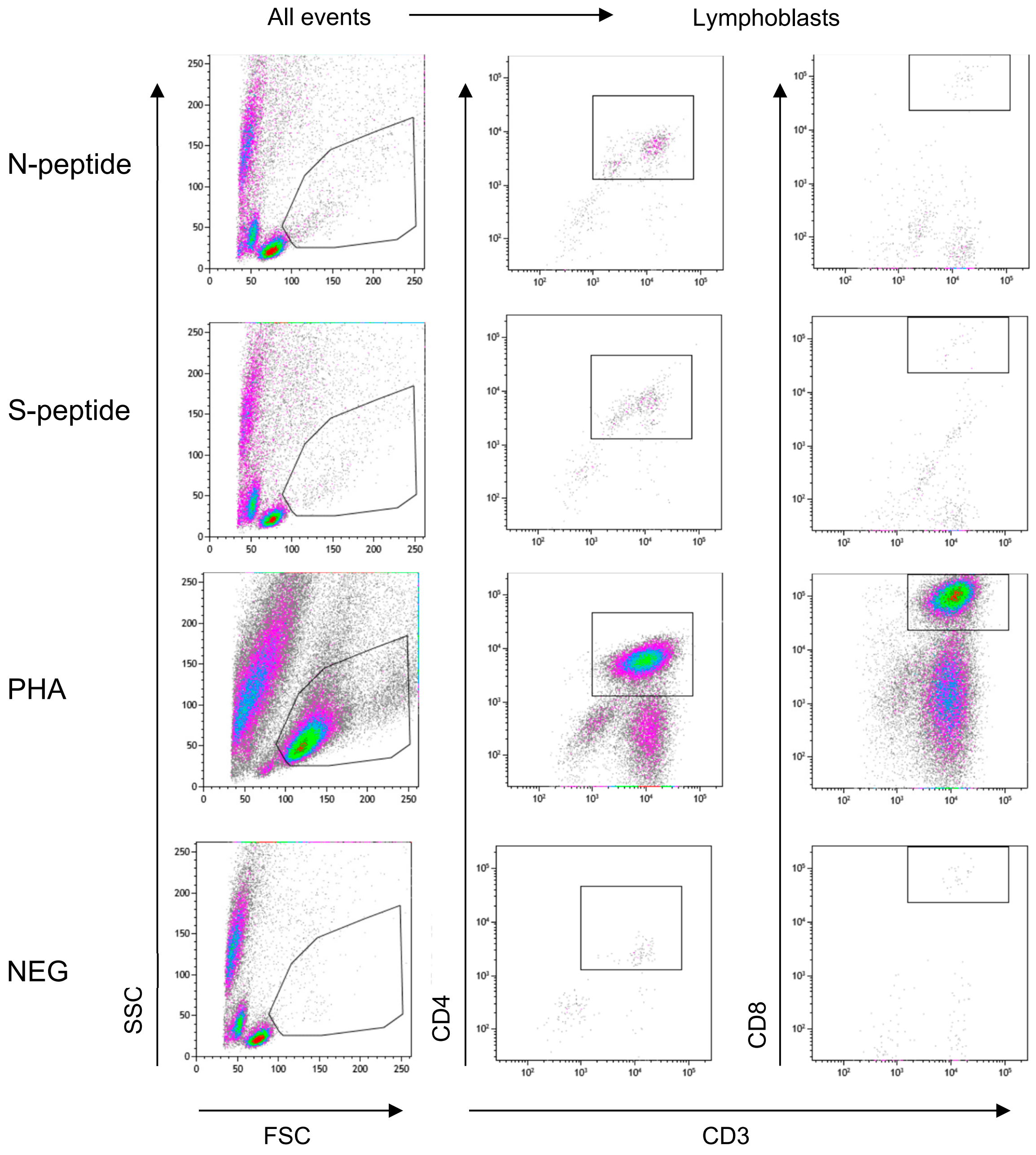

Figure S3. Representative plots of six months sample from an unvaccinated, immunocompetent patient showing the gating strategy of CD4 and CD8 T cell responses to nucleocapsid (N) and spike (S) peptide pools. The mitogen phytohemagglutinin (PHA) was used as positive control for lymphoblast formation and unstimulated whole blood as negative (NEG) control.

Table S1. Immunological profiles of the study cohort at the six-months follow up.

	Reference	Immunocompromised n=6	Immunocompetent n=12	P-value*
Total IgG (g/L) median (range)	6.7-15	9.9 (6.5-57)	9.5 (5.6-15)	0.895
IgA (g/L) median (range)	0.9-4.5	2.1 (0.2-3.4)	1.8 (1.1-7.6)	0.50
IgM (g/L) median (range)	0.27-2.1	0.7 (0.1-1.5)	0.7 (0.1-2.0)	0.835
CD4 T cells (x 10^9/L) median (range)	0.41-1.6	0.57 (0.16-2.9)	0.86 (0.65-1.6)	0.25
CD8 (x 10^9/L) median (range)	0.26-1.0	0.57 (0.10-0.91)	0.73 (0.33-1.5)	0.26
Ratio CD4:CD8 median (range	1.0-2.9	2.1 (0.30-4.5)	1.3 (1.0-2.6)	0.40
B cells (x 10^9/L) median (range)	0.08-0.53	0.18 (0.02-14)	0.24 (0.09-0.62)	0.68
NK cells (x 10^9/L) median (range)	0.11-0.60	0.17 (0.05-0.41)	0.27 (0.16-0.80)	0.13
SARS-CoV-2 vaccination[1]		1 (17)	2 (17)	
SARS-CoV-2 vaccination[2]		2 (33)	5 (42)	0.25
Detected Anti-N IgG n (%)		1 (17)	9 (75)	**0.04**
Detected Anti-S IgG n (%)		6 (100)	12 (100)	>0.999
Detected CD4 anti-S n (%)		4 (67)	12 (100)	0.1
Detected CD8 anti-S n (%)		1 (17)	2 (17)	>0.999
Detected CD4 anti-N n (%)		3 (50)	9 (75)	0.34
Detected CD8 anti-N n (%)		2 (33)	1 (8)	0.24

*Mann-Whitney *U* test for numerical data and Fischer's exact test for binominal data. S, spike protein; N, nucleocapsid protein; [1]one dose; [2]two doses.
